# New Insights on the Fast Response of Poly(Ionic Liquid)s to Humidity: The Effect of Free-Ion Concentration

**DOI:** 10.3390/nano9050749

**Published:** 2019-05-16

**Authors:** Jianxia Nie, Songhua Xiao, Rou Tan, Taihong Wang, Xiaochuan Duan

**Affiliations:** Pen-Tung Sah Institute of Micro-Nano Science and Technology, Xiamen University, Xiamen 361005, China; 17859750523@163.com (J.N.); songhuaxiao@foxmail.com (S.X.); susan.austuin21@yahoo.com (R.T.); thwang@xmu.edu.cn (T.W.)

**Keywords:** poly(ionic liquid)s, humidity sensing, free-ion concentration, fast response and recovery, respiratory rate monitoring

## Abstract

The swelling mechanism is widely used to explain the response of ionic liquids (ILs) or poly(ionic liquid)s (PILs) to moisture. While a fairly broad consensus has been attained, there are still some phenomena that are not well explained. As a complement to the swelling mechanism, we systematically studied the free volume theory in the rapid response and recovery of PIL humidity performance. We chose poly(1-ethyl-3-vinylimidazolium bromide) (PIL-Br), poly(1-ethyl-3-vinylimidazolium tetrafluoroborate) (PIL-BF_4_) and poly(1-ethyl-3-vinylimidazolium bis(trifluoromethane sulfonimide)) (PIL-TFSI) as model materials and investigated the impact of PIL structure including anion type, film thickness and affinity to moisture on performance to obtain the humidity sensing mechanism for PILs based on free volume theory. Hence, we can combine free volume theory with the designed PIL structures and their affinity with moisture to obtain a high concentration of free ions in PIL sensing films. Furthermore, the PIL humidity sensors also show fast, substantial impedance changes with changing humidity for real-time monitoring of the human respiratory rate due to a fast response and recovery performance. Therefore, our findings develop a new perspective to understand the humidity performance of PILs based on free volume theory, resulting in fast response and recovery properties realized by the rational design of PIL sensing films.

## 1. Introduction

The advent of air-stable ionic liquids (ILs) is a milestone in modern analytical science and ILs have been widely used as signal-enhancing elements in electroanalytical applications [1]. As is well known, ILs consist entirely of cations and anions with very specific properties [2], such as high ionic conductivity and negligible vapour pressure, and they have been used in many fields of electrochemical research [3]. In particular, the physical and chemical properties of ILs can be controlled to an unprecedented level through the rational design of cation–anion pairs. Thus, ILs are also known as designer materials or task-specific ILs [4]. Owing to these distinctive features, ILs could be designed and serve as the recognition elements for various sensing platforms [5]. Nevertheless, it should be noted that the direct usage of ILs as sensing materials in their liquid state is inconvenient [6]. It is necessary to immobilize ILs in solid devices for practical applications while keeping their attractive properties [7]. To address this issue, poly(ionic liquid)s (PILs), or polymerized ionic liquids [8], have been studied and represent a class of polymers (or polyelectrolytes) that feature an IL monomer in each repeating unit which is connected through a polymeric backbone to form a macromolecular architecture. Thus, PILs can alleviate the shortcomings, such as leakage and instability, of liquid electrolytes in electrochemical devices [9]. However, the IL moiety is covalently attached to the macromolecule in a PIL which means the organic cation or anion is restricted in mobility. Compared with ILs or IL/polymer mixtures where both the cations and anions are mobile, PILs are considered as single-ion conductors with relatively lower ionic conductivity [10]. Given that the ionic conductivity of PILs has a significant impact on their sensing performance, unfortunately there are relatively few reports on rationalizing the relationship between the PIL molecular structure and its ion transport properties. Therefore, it is truly a big challenge to obtain a fundamental understanding of a molecular-scale framework for designing PILs and its relationship with bulk properties.

PIL responses to chemical species include changes in optical, electric and charge transport properties, each of which have been explored for PIL chemical sensors [11,12,13]. In chemiresistive sensors, PILs are well suited materials as their adjustable conductivity permits a host of well-documented sensing mechanisms. For example, some of the earlier studies utilized PIL-based chemical sensors to detect analytes (such as moisture [14], CO_2_ [15], volatile organic compounds (VOCs) [16], etc.) via a swelling mechanism. In this case, the PIL is well-doped and the analyte is absorbed causing a solvated IL moiety, reducing the viscosity of the PIL and increasing the ionic conductivity, thus creating a reduction in electrical resistance [13]. Under the guidance of this principle, Huang et al. [17] fabricated an optical humidity sensor using PIL photonic crystals as sensing materials. By rational combination of PIL and photonic structure, the optical humidity sensor can be used to rapidly, sensitively and visually detect environmental humidity via a colour change in the whole visible range [18]. To improve the conductivity of PIL-based sensors, single-walled carbon nanotubes [19] and La_2_O_2_CO_3_ nanoparticles [20] were introduced with improved sensing properties because of the synergistic effect. Although the swelling mechanism [21] has been widely applied in IL-based sensors [14], there are still several points that need further improvements. For instance, according to the swelling mechanism, the IL-based humidity sensors often exhibit a fast response property, owing to a strong affinity with water molecules, which usually means that a long recovery time is required. However, this prediction conflicts with our previous experimental results that the PIL-based humidity sensor exhibits both a fast response and recovery properties. Hence, more research is needed for a comprehensive understanding of PIL-based sensors, especially in terms of ionic transport.

Owing to flexibility and easy processing, polymers are considered as attractive sensing materials for humidity sensors, and some polymeric humidity sensors are commercially available [22]. However, conventional polymeric sensing materials, including most neutral polymers or inorganic salt doped polymers, often display poor conductivity due to a low free-ion concentration, resulting in a slow response when exposed to humid conditions. A sub-minute or sub-second response can be achieved by rational design of PIL sensing materials with an adjustable free-ion concentration. Thus, on-line monitoring of humidity is expected to be realized and applied to various healthcare areas [23], such as pulmonary-function diagnostics [24], management of patients undergoing anaesthesia and critical care medicine [25]. In this regard, we systematically investigated the humidity sensing performance of imidazolium-based PIL films. We combine the free volume theory with the designed PIL structures including anion types, film thickness and affinity with moisture to obtain a high concentration of free-ions in PIL sensing films. Thus, the conductivity of PIL humidity sensors was found to vary sensitively over a wide range of relative humidity levels (RH) with a fast response and recovery properties. Furthermore, the PIL humidity sensors also confirmed a fast, substantial impedance change with a change in humidity for real-time monitoring of the human respiratory rate due to their fast response and recovery performance. Therefore, our findings develop a new perspective to understand the humidity performance of PILs based on free volume theory, resulting in a fast response and recovery properties realized by the rational design of PIL sensing films. It is highly expected that the PIL humidity sensors can be implemented for continuous monitoring of humidity in various arenas.

## 2. Materials and Methods

### 2.1. Chemicals and Reagents

1-vinyl-3-ethylimidazolium bromide (VEIm-Br) was obtained from Lanzhou Greenchem ILS, LICP, CAS, China. Then, 2,2′-azobis(2-methylpropionitrile) (AIBN), sodium tetrafluoroborate (NaBF_4_) and lithium bis(trifluoromethane sulfonimide) (LiTFSI) were purchased from the Sigma-Aldrich Company (Shanghai, China) and were used as received without further purification. The water that was used was deionized.

### 2.2. Synthesis of Poly(1-ethyl-3-vinylimidazolium bromide) (PIL-Br)

In a typical synthesis process, 10.38 g of 1-vinyl-3-ethylimidazolium bromide, 30 mg of AIBN and 70 mL of absolute ethanol were added into a 250 mL round-bottom flask. The mixture solution was stirred for 2 h under nitrogen atmosphere and was then heated to 70 °C for 24 h. After that, the resulting PIL was washed with tetrahydrofuran (THF) and then dried at 70 °C overnight. The schematic diagram is as follows:

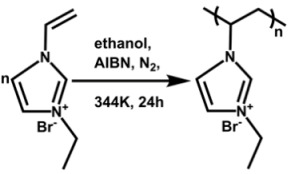



### 2.3. Synthesis of Poly(1-ethyl-3-vinylimidazolium tetrafluoroborate) (PIL-BF_4_)

In this synthesis process, 2.04 g of PEVIm-Br was dissolved in a mixed solution composed of 9 mL of deionized water and 9 mL of absolute ethanol (solution A) under magnetic stirring in an ice-water bath for 30 min. Then, 1.10 g of NaBF_4_ was dissolved in another mixed solution composed of 9 mL of deionized water and 9 mL of absolute ethanol (solution B) under magnetic stirring in an ice-water bath for 30 min. Subsequently, solution B was slowly added into solution A and then the mixture was stirred in an ice-water bath for 30 min and was left to stand for 1 h. Finally, the resulting yellowish white solid was cleaned with deionized water and absolute ethanol several times before drying at 70 °C for 24 h. The schematic diagram is as follows:

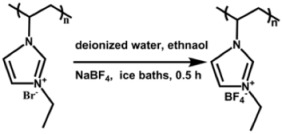



### 2.4. Synthesis of Poly(1-ethyl-3-vinylimidazolium bis(trifluoromethane sulfonimide)) (PIL-TFSI)

The protocol for the synthesis of PIL-TFSI is similar to that of PIL-BF_4_: 2.04 g of PIL-Br reacts with 2.81 g of bis(trifluoromethane sulfonimide) through an ion exchange reaction and the resulting yellowish solid was finally gained. The schematic diagram is as follows:

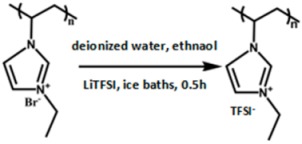



### 2.5. Characterization

The as-prepared PILs were characterized by Fourier-transform infrared spectroscopy (FTIR), ^1^H NMR spectra, etc. TMDSC (NETZSCH DSC 204 F1) was used to determine the *T_g_* of PIL-Br equilibrated at varying RH levels under nitrogen. First, a 250 mL bottle containing saturated aqueous solutions of K_2_SO_4_ salts was used to equilibrate PIL-Br for 2 h. Then, the sample was placed into a non-hermetically sealed aluminium differential scanning calorimeter (DSC) pan for measurement. The TMDSC runs consisted of cooling at a rate of 5 °C/min to −60 °C, followed by heating to 100 °C (with the same heating rate of 5 °C/min) and a temperature modulation of ±0.80 °C every 60 s. The DSC heats from 30 to 250 °C at a heating rate of 10 °C/min.

### 2.6. Humidity Sensor Preparation and Measurements

The humidity sensors were fabricated by spin-coating the PIL dispersion onto pre-cleaned interdigitated electrodes. Alumina ceramic was used as the substrate due to its high dielectric strength and excellent stability. Interdigitated electrodes composed of 10 nm of titanium and 80 nm of gold with a linewidth of 80 μm were fabricated by sputtering, as shown in Figure S1.

Spin-coating PIL onto the interdigitated electrodes: 0.06 g of PIL-Br was added into 600 mL of absolute ethanol and the mixture solution was then stirred for 2 h until completely dissolved. The sensors were fabricated by spin coating 4.0 μL of the mixture solution onto the pre-cleaned interdigitated electrodes at a speed of 4000 rad/s for 60 s. After that, the sensor was dried at room temperature for 48 h. For the case of PIL-BF_4_, the solution was replaced by *N,N*-dimethylformamide (DMF) with the other conditions preserved to be the same. The PIL-Br thickness can be easily tuned by adjusting the solution concentration and revolving speed.

Humidity sensing properties were tested by an impedance analyser (Agilent 4294, 40 Hz to 110 MHz). The applied voltage amplitude was 500 mV and the measuring temperature was approximately 25 °C. Several 250 mL bottles containing different saturated salt solutions including LiCl, MgCl_2_, K_2_CO_3_, NaBr, NaCl, KCl and K_2_SO_4_ were used as the sources of humidity for 11%, 33%, 43%, 59%, 75%, 85% and 98% RH, respectively.

## 3. Results and Discussion

### 3.1. Structural Characterization of PILs

**Figure 1:**
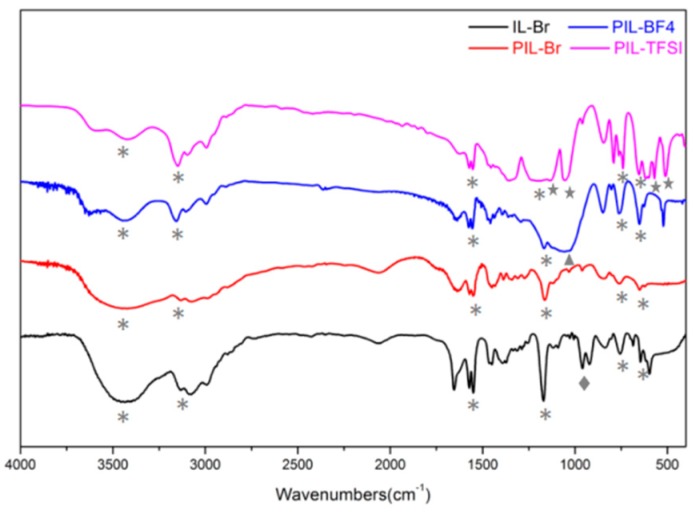
FT-IR spectra for IL-Br, PIL-Br, PIL-BF_4_ and PIL-TFSI.

The obtained PILs were first characterized by FTIR and ^1^H NMR. The FT-IR spectra for the IL and PILs are shown in Figure 1 and the absorption peaks for the imidazolium rings in the IL and PILs are obvious. The C−H bonds in the imidazolium rings show a deformation vibration in-plane peak at 1170 cm^−1^ and external bending vibration peaks at ~750 cm^−1^, ~650 cm^−1^. The absorption peak at ~3140 cm^−1^ arises from the stretching vibration of the C−H bonds, and that at ~1570 cm^−1^ is due to the C=N bonds in the imidazolium rings. Thus, the results from the FT-IR spectra can clearly confirm the integrity of the imidazolium ring in the PILs. However, a complete disappearance of the band corresponding to Br^−^ (960 cm^−1^) is observed with the appearance of new bands attributed to BF_4_^−^ (1053 cm^−1^) or TFSI^−^ (~1187 cm^−1^), which also proves the success of the anion exchange. In addition, the absorption peak at 3450 cm^−1^ is attributed to the hydroxyl groups (H−O) of water molecules mechanically adsorbed by the IL or PILs.

**Figure 2:**
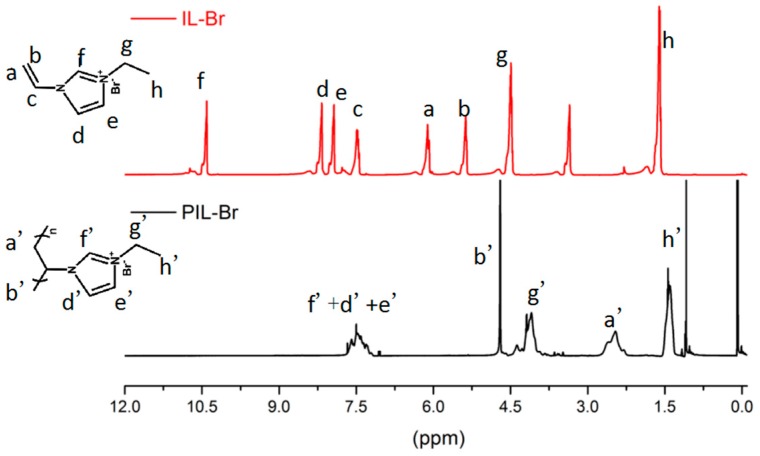
600-MHz ^1^H NMR spectra for PIL-Br and IL-Br.

The ^1^H NMR spectra for PIL-Br and IL-Br is shown in Figure 2 and the chemical formulae are shown in the illustration. The vinyl signals at 7.5, 6.1 and 5.4 ppm are observed to disappear, with the appearance of new signals at 2.7 and 4.5 ppm attributed to the protons of the polymeric backbone. Under the influence of the chemical group, the transformation and shift of signals near 10.5 and 8 ppm are distinct. We ensure that the polymerization by IL-Br monomer is successful via ^1^H NMR test.

**Figure 3:**
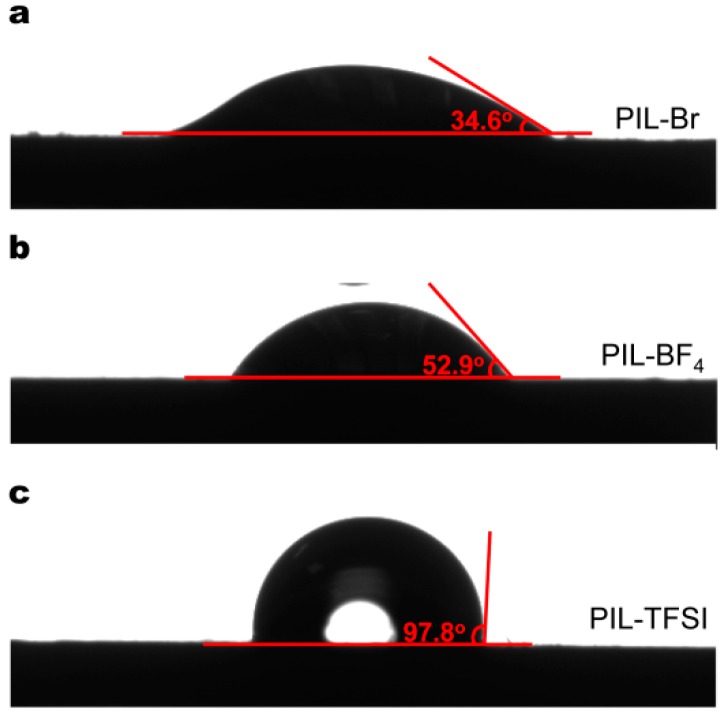
Static water contact angles corresponding to the as-prepared poly(ionic liquid)s (PILs): (**a**) PIL-Br, (**b**) PIL-BF_4_ and (**c**) PIL-TFSI.

Wetting, usually associated with hydrophilicity, is a phenomenon based on two-phase interface changes from a solid-gas interface to a solid-liquid interface. The wettability of a film material can be expressed by the contact angle (1–180°) and a large contact angle indicates a worse wettability. The static water contact angles for PIL-Br (34.6°), PIL-BF_4_ (52.9°) and PIL-TFSI (97.8°) are shown in Figure 3, revealing the hydrophilicity: PIL-Br>PIL-BF_4_>PIL-TFSI. The result corresponds to the effect of the anionic polarity to water molecules in theory.

### 3.2. Swelling Mechanism for a Hydrophilic PIL-Br Humidity Sensor: The Effect of Thickness

**Figure 4:**
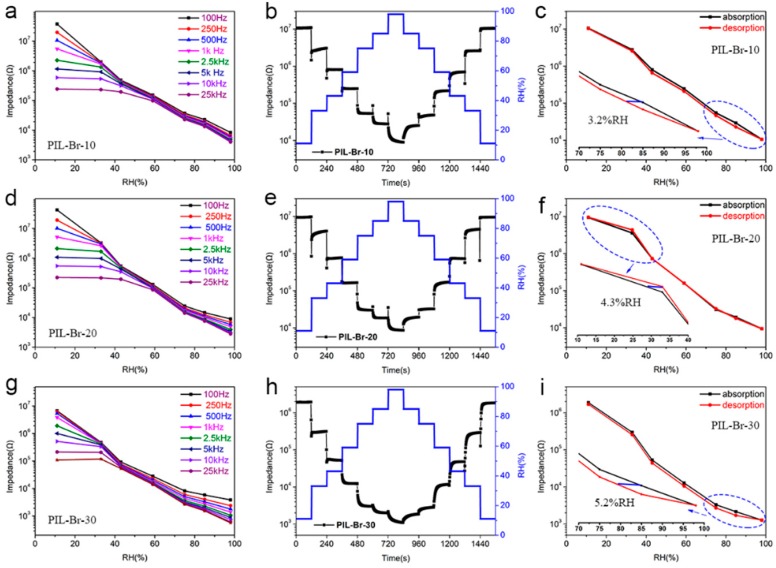
Thickness-dependent performance of PIL-Br humidity sensors: the impedance at varying RH levels measured under various frequencies: (**a**) PIL-Br-10, (**d**) PIL-Br-20, (**g**) PIL-Br-30, respectively; the dynamic response of PIL-Br sensors for increasing RH from 11% to 98% RH followed by a return to the initial state: (**b**) PIL-Br-10, (**e**) PIL-Br-20, (**h**) PIL-Br-30, respectively; the humidity hysteresis of (**c**) PIL-Br-10, (**f**) PIL-Br-20 and (**i**) PIL-Br-30, respectively.

When polymers are used in humidity sensing, the swelling process is usually exploited: (i) diffusion of water molecules through the polymer matrix, (ii) relaxation of polymer chains and (iii) expansion of the polymer network upon relaxation. To confirm that the humidity performance of the PILs can be affected by their swelling behaviour, a series of controllable experiments were exemplified using hydrophilic PIL-Br. Since the swelling behaviour of a PIL sensing film is directly related to its thickness, we thus investigate the effect of PIL-Br thickness on the humidity performance. The thickness of PIL-Br sensing films can be easily controlled by adjusting the concentration of the spin-coating solution, denoted as PIL-Br-10, PIL-Br-20 and PIL-Br-30, respectively. It is well known that the working frequency has an important impact on the humidity performance of a PIL sensor. To explore the optimum frequency for the PIL humidity sensors, we first investigated the impedance as a function of RH at different frequencies. The relationships between the impedance modulus of PIL-Br-10 and RH with measuring frequency from 100 Hz to 25 kHz are shown in Figure 4a. In the low frequency region from 100 Hz to 1 kHz, the PIL-Br-10 sensor presents good linearity with a total impedance modulus change of more than two orders of magnitude, which indicates high sensitivity in the whole RH range from 11% to 98% RH. However, the impedance curve gradually turns flat and the total impedance modulus change is reduced when the measuring frequency is higher than 2.5 kHz. This is mainly because the polarization of the water molecules cannot catch up with the directional change of the electrical field under high frequency and only electronic-displacement polarization and ion-displacement polarization can take place. Since the capacitance and dielectric constant of the PIL sensor is very small, it becomes independent of RH at high frequencies. The best linearity of the impedance versus RH curve for the PIL-Br-10 sensor appears at 500 Hz; thus, 500 Hz was defined as the ideal working frequency for the PIL-Br-10 sensor and was used in the following measurements. Similarly, the optimum measuring frequencies for PIL-Br-20 and PIL-Br-30 can be calculated to be 500 Hz (Figure 4d) and 2.5 kHz (Figure 4g), respectively. The dynamic impedance response of PIL-Br sensors within the step changes from 11% to 98% RH followed by a return to the initial state were also performed, as shown in Figure 4b,e,h. All sensors exhibited a good reversibility during the step change in the humidity levels, revealing good reliability of the as-prepared humidity sensors. In addition, humidity hysteresis—another criterion that is commonly used to estimate the reliability of humidity sensors—was also tested for PIL-Br sensors with varying thickness. The humidity hysteresis is defined as the maximum difference of the humidity sensor between the adsorption and desorption process [26]; thus, the humidity hysteresis was calculated to be 3.2% RH (Figure 4c), 4.3% RH (Figure 4f) and 5.2% RH (Figure 4i) for PIL-Br-10, PIL-Br-20 and PIL-Br-30 within the working range of 11%–98% RH, respectively. This result can further confirm the good reliability of PIL-Br humidity sensors.

**Figure 5:**
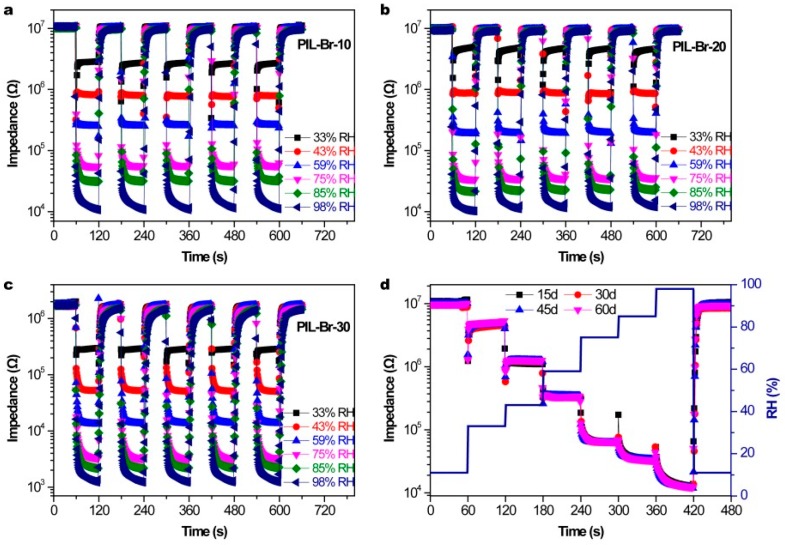
The repeatability characteristic of PIL-Br sensors from 11% to 98% RH: (**a**) PIL-Br-10, (**b**) PIL-Br-20 and (**c**) PIL-Br-30, respectively, (**d**) Long-term stability of a PIL-Br-10 humidity sensor.

**Figure 6:**
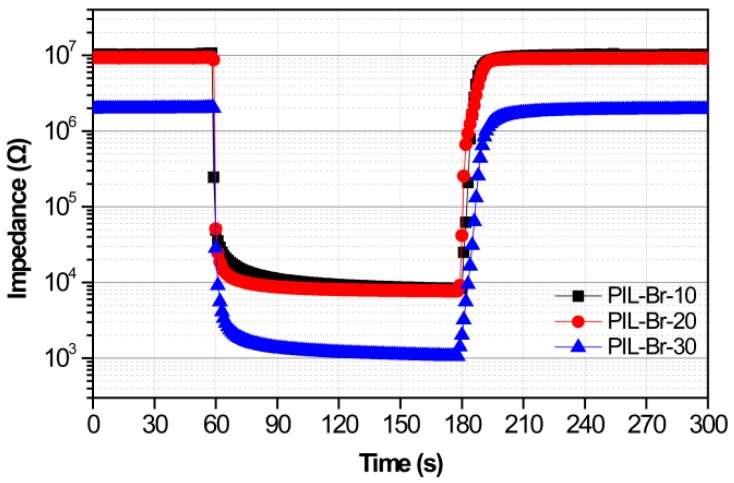
Response and recovery properties for PIL-Br humidity sensors with varying thickness.

The repeatability of PIL-Br sensors was further characterized by switching the measurement environments between 11% RH and various RH for five cycles, as shown in Figure 5a–c. All the PIL-Br sensors with varying thicknesses possess good repeatability. At the same time, the response and recovery times of these sensors were characterized in Figure 6. The sensor with the thinnest film has pretty short response and recovery times (6 s/10 s). In addition, we wondered the differences between these PIL-Br sensors in long-term stability, so we tested these sensors repeatedly per 15 days under the fixed humidity levels. The PIL-Br-10 sensor can maintain quite a stable dynamic response, as shown in Figure 5d. The data show good consistency of the PIL-Br-10 sensor at each humidity region. These results demonstrate that the as-prepared PIL-Br-10 sensors possess high stability and durability in a wide RH range. In contrast, the long-term stabilities of two other sensors which have thicker film are not good enough. This can be seen in Figure S2.

**Figure 7:**
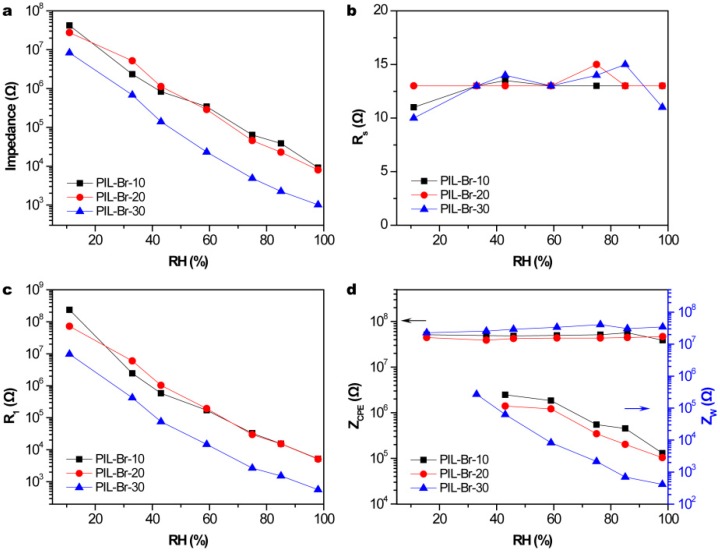
Dependence of the values of (**a**) total, (**b**) *Rs*, (**c**) *R*_1_ and (**d**) *Z_CPE_* and Z_W_ on varying RH levels for PIL-Br sensors with different thicknesses.

For further discussion, the experimental impedance results obtained for the PIL-Br humidity sensors were fitted via equivalent circuits by using Z-View software, as shown in Figure 7. Here, *R_S_* is related to the resistance of the electrode/film, *R*_1_ is the bulk resistance of film, *Z_CPE_* is a constant phase element (CPE) and *Z_W_* is a Warburg impedance [27]. As shown in Figure 7a, the total impedance of the PIL-Br humidity sensors almost achieves a three orders of magnitude response and superior linear correlation coefficient under RH ranging from 11%–98%, which is advantageous for humidity sensors. The values for *R_S_* and *Z_CPE_* were hardly affected by ambient humidity, as shown in Figure 7b,d, because *R_S_* relates only to the inter-contact between the electrode and film and *Z*_CPE_ is characterized as the influence of polarization in the sensing film. In addition, *R_W_* decreases drastically as RH increases from 43% to 98%, leading to high-concentration and fast-action of mobile ions in the diffusing process due to the formation of continuous multi-water layers at 33%RH(PIL-Br-30) or 44%RH(PIL-Br-10, PIL-Br-20). It is worth noting that continuous multi-water layers can promote jumping of hydronium ions (H_3_O^+^) and free hydrogen ions (H^+^) for conduction based on the Grotthuss mechanism [28]. Hydrogen-ion jump conduction occurs when a H^+^ (H_3_O^+^) passes from one end to the other through an adjacent water molecule, rather than a H^+^ (H_3_O^+^) going from one end to the other, with the former being faster. The value of *R*_1_ decreases linearly by approximately three orders of magnitude within the full measured RH range (Figure 7c), which can be attributed to the improved conductance of the PIL-Br sensing film because of the increased number of absorbed water molecules. In addition, the results show that it is easy to increase the conductivity in thicker films because of the absorption of more water molecules. In summary, the PIL-Br-30 sensor has pretty outstanding conductivity during the ion diffusion process.

### 3.3. Humidity Mechanism for PILs Based on Free Volume

As mentioned above, PILs are usually considered to be single-ion conductors that are comprised of polymeric imidazolium-based cations and anions. In these cases, cations are structurally constrained as part of the polymer skeleton, which largely restricts their movement. Owing to the asymmetric volume of the cation and anion, there are large spaces (i.e., holes and voids) in the PILs. These spaces are also denoted as free volume, which can provide sorption sites to accommodate penetrate of water molecules and to allow ion-movement through the PIL matrix [29]. The permeation of water molecules through the free volume can induce a swelling of the PILs, thus altering their properties. Kasapis et al. [30] reported the swelling behaviour of polymers exposed to a hot and humid environment. The hygroscopic swelling of a polymer can cause a change in volume, similar to the thermal expansion caused by an increase in temperature, leading to a linear expansion of the free volume (*f*) of the polymer. Following derivation from the Williams-Landel-Ferry (WLF) expression [31], the time-temperature-humidity equivalence principle can be expressed as follows:

(1) $$f=f_{0}+e_{T}\left(T-T_{0}\right)+e_{M}\left(M-M_{0}\right)$$

where *f* is the free volume of a polymer, *f*_0_ is the free volume fraction of a sample at the reference temperature *T*_0_; $e_{T}$ and $e_{M}$ is the free volume fraction of the thermal expansion coefficient and humidity swelling coefficient, respectively; *M*_0_ is the water content of a sample at the reference temperature *T*_0_. Assuming that a sample remains in the same ambient temperature, the free volume will increase as the ambient humidity increases. According to the free volume theory, the relationship between the viscosity (*η*) and the free volume (*f*) of the polymer follows the Doolittle equation [32]:

(2) $$\ln\eta =\ln A+B\left[\frac{1}{f}-1\right]$$

where *A* and *B* are constants for a given material. Thus, the viscosity of PILs will decrease as the free volume increases. Furthermore, the relationship between conductivity (*η*) and viscosity (*σ*) of the PILs can be built by the following Walden equation:

(3) $$\mathrm{lg}\sigma =\mathrm{lg}k+\alpha \mathrm{lg}\eta^{-1}$$

where *k* is a temperature dependent constant and *α* is a fitting parameter [33]. Thereby, the conductivity in PILs is inversely proportional to the viscosity and proportional to the free volume. Therefore, the conductivity of the PIL sensing films can be optimized by adjusting their free volume, which is conducive to the design of sensing materials for better performance.

**Figure 8:**
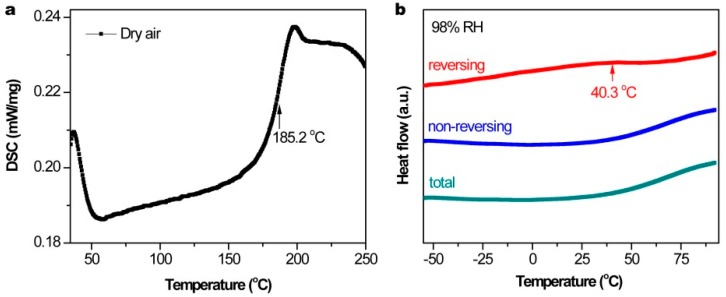
(**a**) DSC thermogram of PIL-Br equilibrated under dry conditions. (**b**) TMDSC thermogram of PIL-Br equilibrated under 98% relative humidity levels (RH): total, reversing and non-reversing heat-flow signals.

Generally, the glass transition temperature (*T_g_*) is defined as the temperature at which the mechanical properties of a polymer material radically change due to internal movement of the polymer chains [34]. For the as-prepared PILs, a lower *T_g_* promotes a higher ionic conductivity. To verify that the PIL conductivity changes with a change in humidity based on free volume theory, we take PIL-Br as an example and investigate its *T_g_* change under varying RH levels using temperature-modulated differential scanning calorimetry (TMDSC). From Figure 8a, the *T_g_* of PIL-Br under dry conditions was measured to be 185.2 °C according to a DSC scan. For comparison, a typical TMDSC scan obtained from PIL-Br equilibrated to 98%, as shown in Figure 8b. The endotherm of the total signal at approximately 88 °C, which corresponds to the evaporation of the absorbed water, is exclusively present in the non-reversing heat-flow signal. The inflection point in the reversing signal at 40.3 °C occurs at a temperature that is well below the onset of water evaporation and designates the *T_g_* of PIL-Br under a 98% RH condition. Thus, the *T_g_* of PIL-Br decreases with increasing RH, which benefits improved ion movement. According to the Vogel-Tamman-Fulcher (VTF) equation: $\sigma =\sigma_{0}e^{-B/T-T_{0}}$, where σ  is the conductivity, $\sigma_{0}$ and  B are constants and $T_{0}$ is the Vogel temperature where ion motion first occurs ($T_{0}=T_{g}-50\;K$) [35]. Therefore, the conductivity of PIL-Br increases under a high RH condition, which is well consistent with our hypothesis.

### 3.4. The Effect of Anion Type on Humidity Sensing Performance of PIL

**Figure 9:**
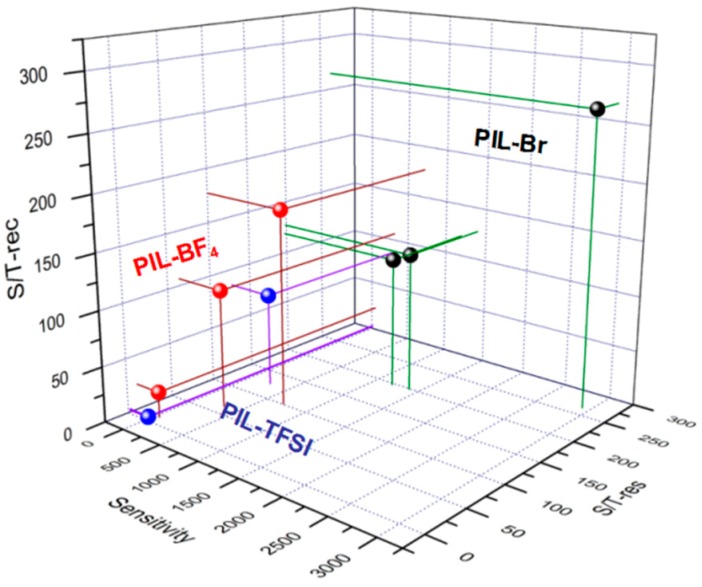
Response of sensors based on PILs(PIL-Br, PIL-BF_4_ and PIL-TFSI) at the working frequency.

Molecules with polar groups, which have a great affinity with water, can attract water molecules or be dissolved in water easily. Furthermore, the excellent hydrophilic PILs can absorb a large volume of water to increase their free volume according to the WLH equation. Thus, it is interesting to study the sensing performance of different hydrophilic PILs films. Adjusting the anion type in PILs film is an effective route to change free volume. A series of controllable experiments were designed using PILs with different anions to investigate the effect of anion type on humidity performance. PIL-BF_4_ and PIL-TFSI were applied as humidity materials in our work because of their different hydrophilic performances. The method used to spin-coat PIL onto the interdigitated electrodes is similar to that used for the humidity sensor based on PIL-Br, as shown in Supporting S1. The humidity performance of the PIL sensors was tested at the working frequency, as shown in Figures S3–S8. To clearly observe the effect of anion type, we list the performance parameters for the PIL-based humidity sensors in Table 1. Here, *S*/*T-res* is defined as the ratio of the sensitivity to response time and, similarly, *S*/*T-rec* is defined as the ratio of the sensitivity to recovery time. These ratios are perfect for reflecting the velocity for response or recovery, especially for a response with different orders of magnitude. Figure 9 shows the performance of the PIL-based humidity sensors, with data far away from the origin of the coordinates representing high sensitivity, high response and recovery velocity. The different level of performance among the three PIL-based humidity sensors could be clearly distinguished, with the PIL-Br humidity sensors showing an obviously higher sensitivity, response and recovery velocity.

The PIL structure is special because of the many three-dimensional spaces which are due to the staggered connections between the polymer chains, similar to an overpass-type structure. Meanwhile, these three-dimensional spaces are conducive to increase the free volume by absorbing water molecules for current carrier transport. At this basis, the synergistic effect of three-dimensional space and hydrophilic PIL can significantly improve the humidity performance of sensors. For example, the PIL-based humidity sensors exhibit varying sensitivity from 11% to 98% RH at room temperature (25 °C), as shown in Figure 9. Based on the static water contact angle, the hydrophilicity follows the order PIL-Br > PIL-BF_4_ > PIL-TFSI. Among the PILs in our work, the PIL-Br sensor which has the optimum hydrophilicity possesses a biggest free volume based on the swelling theory. Fortunately, the larger free volume can provide more absorption sites to make water molecules permeate into the PIL film for more carrier transport channels at the same ambient humidity and temperature. Due to the electrostatic fields of the PIL-Br film, the absorbed water molecules will dissociate as 2H_2_O → H_3_O^+^ + OH^−^. In addition, the conduction in polymer films mainly occurs along the hydrogen bond via hydrogen-ion jumps based on the Grotthuss mechanism, i.e., H_2_O + H_3_O^+^ → H_3_O^+^ + H_2_O. Thus, the PIL-Br film has more free ions and carrier transport channels to achieve outstanding conductivity, high sensitivity and a large response and recovery velocity, which is verified by our experimental data.

### 3.5. Possible Mechanism for the Fast Response and Recovery of PILs

**Figure 10:**
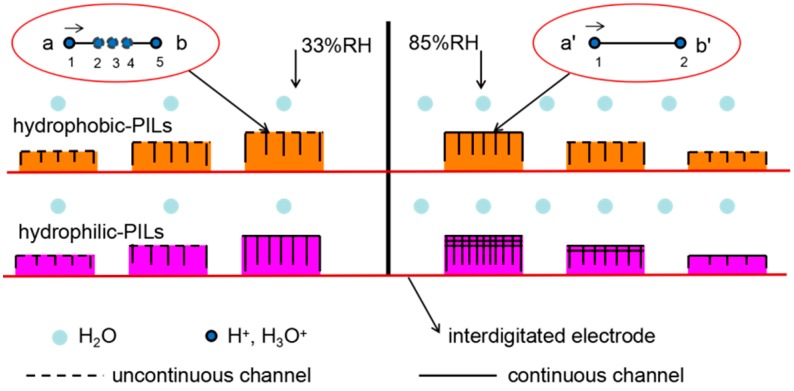
Schematic illustration of the humidity sensing mechanism for a PIL film with varying thickness and anion type.

As mentioned above, the conductivity of the PIL is inversely proportional to the viscosity and proportional to the free volume through the free volume theory. At the same time, the free volume of PIL has affinity with moisture. Therefore, the mechanism of the fast response and recovery of the PILs can be explained by the free volume theory, as shown in Figure 10. The response process of the PIL-based humidity sensor is described from low RH to high RH as follows: (i) increase of the free volume of PIL film through combining with water molecules to provide more carrier transport channels, (ii) obtain a high concentration of free ions because of the relaxation and disintegrate effect of the polymer chains and dissociation of the absorbed water molecules and (iii) increased conductivity in a PIL film because of the fast free-ion movements in the channels. The PIL structure including anion types and film thickness affected the free volume of the PIL film at the same ambient humidity at 25 °C; then, the hydrophilic anion and slightly thick film benefits an increase in the free volume. In addition, the magnitude of the free volume of the PIL film can decide the lowest humidity required to achieve continuous multi-water layers for providing more carrier transport channels. Meanwhile, the concentration of free ions including H^+^, H_3_O^+^, Br^−^ and BF_4_^−^ can increase as the ambient humidity increases. Therefore, our findings develop a new perspective to understand the humidity performance of PILs based on free volume theory and we can rationally design PIL structures including anion types and film thickness to obtain super properties including fast response and recovery in PIL sensing films.

### 3.6. Real-Time Monitoring of Human Respiratory Rate

Human breath is a highly complex mixture of more than 100 types of gases, many of which can provide useful information in the monitoring of the human health condition [36]. Water vapour—one of the main ingredients of human breath—is easily detected by a humidity sensor and the change in humidity for respiratory airflow can be used to effectively evaluate human health status. Nowadays, the increase in the incidence of sleep apnea syndrome (SAS) has aroused people’s attention to the monitoring of human sleep and has led to a large number of related studies. Apnea during sleep may cause serious consequences and even death. However, this situation will be ameliorated if the sleep apnea of a human is found in a timely manner and appropriate rescue measures are taken.

**Figure 11:**
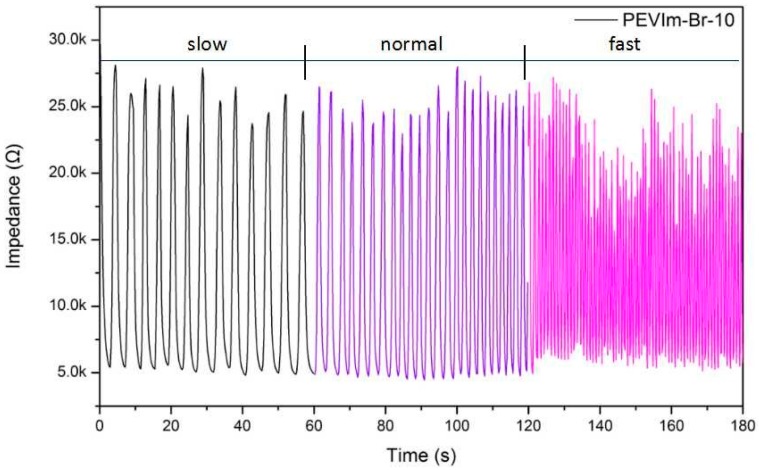
Response of the sensor based on PIL-Br-10 at varying respiratory rates at the working frequency (500 Hz).

In recent years, great breakthroughs have been made in research on human respiration monitoring. For example, Yun-Ze Long et al. [37] prepared 1D sensing materials by electrospinning, which were utilized as smart fabrics for avoiding sleep apnea. Youju Huang et al. [38] fabricated a hybrid PNIPAm/AuNP aerogel-based humidity sensor and used it to detect human breath in different states or from different individuals for human health monitoring. Jin Zhou et al. [39] prepared humidity sensors based on a polysquaraine, poly(1-phenylpyrrole-2-ylsquaraine) (PPPS) loaded with varying amounts of Au nanoparticles, which were applied to human respiratory monitoring, showing excellent moisture sensitivity and practicality. In our work, the humidity sensor based on PIL-Br-10 has superior performance, including a fast response, high sensitivity and excellent long stability, and it can be applied in the real-time monitoring of the human respiratory rate. The PIL-Br-10 film could easily detect the change in humidity in respiratory airflow, which could effectively evaluate human health status. Hence, we put the humidity sensor based on the PIL-Br-10 into a mask to fabricate a breath mask, as shown in Figure S9; the sensor was placed at a distance of 3.5 cm from the nose and 2.5 cm from the mouth of a tester. A volunteer (healthy female) wore the breath mask to breathe in a fast, normal or low rate, and the testing results are shown in Figure 11. We can obviously see the distinction for breathing fast, normally or at a low rate, even obtaining a breathing period between 0.9 to 4.3 s, as shown in Figure 11**;** the frequency of the curve varies with the rate of respiration, revealing that our PIL-Br-10 sensor can capture the signals of humidity change under different rates. This proves that the sensor has practicable application for real-time monitoring of human respiratory rates.

## 4. Conclusions

In summary, PILs were successfully synthesized by typical polymerization and an anion exchange method and were used as humidity materials. We studied the effects of the PIL structure including film thickness and anion type on PIL-based humidity sensor performance. The results showed that a hydrophilic anion and slightly thick film can benefit an increase in the free volume of PIL films to obtain a high concentration of free ions and effective carrier transport channels. Furthermore, the humidity sensing mechanism of PILs was superbly explained by the free volume theory. It is worth noting that our work develops a new perspective to understand the humidity performance of PILs based on free volume theory, resulting in fast response and recovery properties realized by the rational design of PIL sensing films. In our work, the PIL-Br-10 humidity sensor shows high sensitivity (1190), small hysteresis (3.2%), enhanced durability and rapid response (6s)/recovery (10s). We also apply the PIL-Br-10 sensor to monitor human breath under varying conditions, revealing excellent performance and practicability values. Therefore, the PIL-based sensor with novel and reasonable design is highly promising for a broad range of applications.

## Figures and Tables

**Table 1:** The performance parameters of the as-prepared PIL-based humidity sensors.

Sensor	Sensitivity	Response Time (s)	Recovery Time (s)	S/T-res	S/T-rec
PIL-Br-10	1190	6	10	198.3	119.0
PIL-Br-20	1392	7	11	198.9	126.5
PIL-Br-30	2916	11	11	265.1	265.1
PIL-BF_4_-10	46	4	2	11.5	23.0
PIL-BF_4_-20	319	5	3	63.8	106.3
PIL-BF_4_-30	708	7	4	101.1	177.0
PIL-TFSI-10	4	2	2	2.0	2.0
PIL-TFSI-20	9	4	3	2.3	3.0
PIL-TFSI-30	254	2	3	127.0	84.7

## Supplementary Materials

The following are available online at https://www.mdpi.com/2079-4991/9/5/749/s1.
