# Supplementary material for: New Insights on the Fast Response of Poly(Ionic Liquid)s to Humidity: The Effect of Free-Ion Concentration
Source: Nanomaterials (Basel). 2019 May 16;9(5):749. doi: 10.3390/nano9050749 (PMC6567128; doi:10.3390/nano9050749)
Supplement: Supplementary file 1 [file nanomaterials-09-00749-s001.pdf]

Supplementary Information

New Insights on the Fast Response of Poly(Ionic Liquid)s to Humidity: The Effect of Free-Ion Concentration

Jianxia Nie †, Songhua Xiao †, Rou Tan †, Taihong Wang and Xiaochuan Duan *

Pen-Tung Sah Institute of Micro-Nano Science and Technology, Xiamen University, Xiamen, 361005, China; 1838252519@qq.com (J.N.), songhuaxiao@foxmail.com (S.X.), 1152951140@qq.com (R.T.), thwang@xmu.edu.cn (T.W.)

* Correspondence: xcduan@xmu.edu.cn

Supporting S1: Spin-coating poly(ionic liquid)s (PIL) onto the interdigitated electrodes: 0.06 g of PIL-Br was added into 600 mL of solvent, and then the mixture solution was stirred for 2 h to be completely dissolved. The sensors were fabricated by spinning 4.0 μL mixture solution onto the pre-cleaned interdigitated electrodes at the speed of 4000 rad/s for 60 s. For the case of PIL-BF_4, the solution was replaced by *N*,*N*-dimethylformamide (DMF), and for PIL-TFSI, the solution was replaced by acetone with other conditions preserved the same. The thickness of PIL can be easily tuned by adjusting the solution concentration and revolving speed.

Table S1. The contents of the as-prepared poly(ionic liquid)s (PILs) and solvent.

Sensor	PIL-Br (g)	Ethanol (mL)	Sensor	PIL-BF4 (g)	DMF (mL)	Sensor	PIL-TFSI (g)	Acetone (mL)
PIL-Br-10	0.06	600	PIL-BF4-10	0.06	600	PIL-TFSI-10	0.06	600
PIL-Br-20	0.12	600	PIL-BF4-20	0.12	600	PIL-TFSI-20	0.12	600
PIL-Br-30	0.18	600	PIL-BF4-30	0.18	600	PIL-TFSI-30	0.18	600

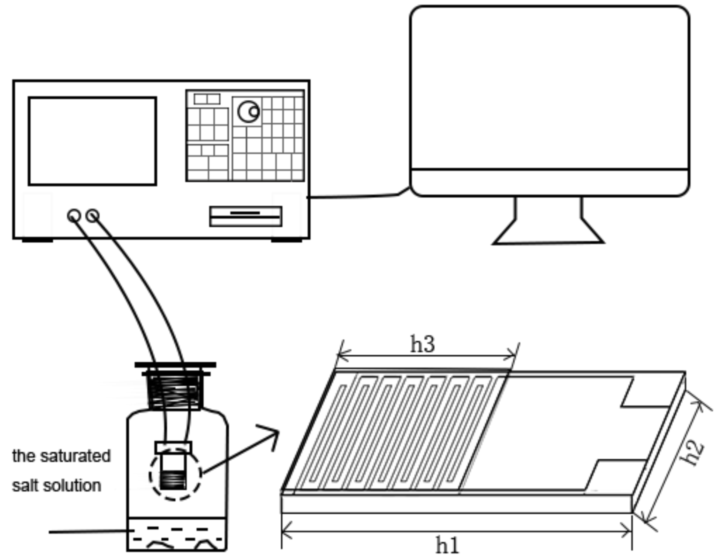

Figure S1. The structure of the interdigitated electrodes and the testing process of the humidity sensor based on the as-presented PILs.

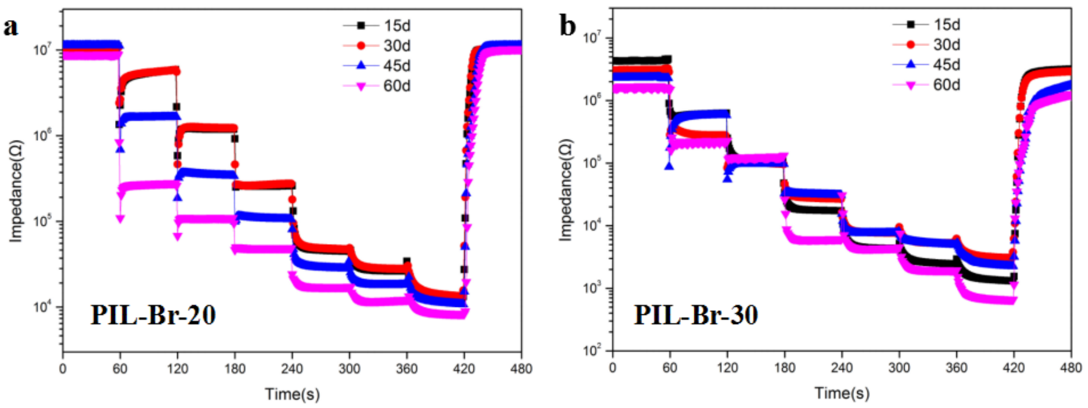

Figure S2. Long-term stability of the PIL-Br-20 and PIL-Br-30 humidity sensor.

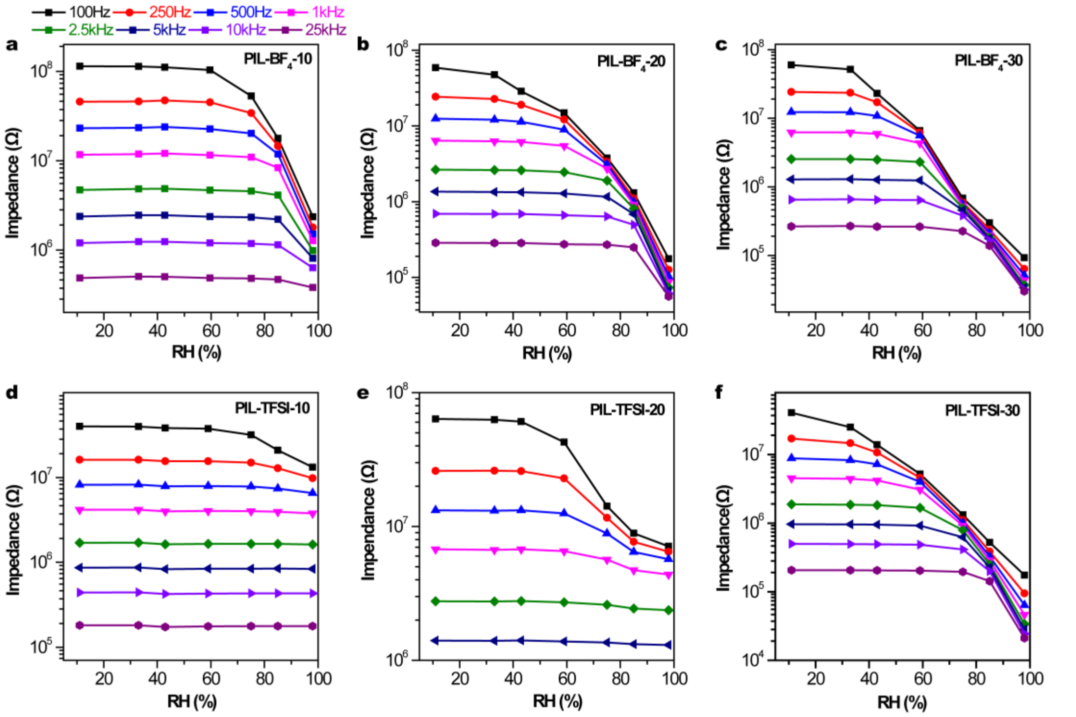

Figure S3. Thickness-dependent performances of PIL-BF_4 and PIL-TFSI humidity sensors: the impedance at different relative humidity levels (RH) measured under various frequencies: (a) PIL-BF_4-10, (b) PIL-BF_4-20, (c) PIL-BF_4-30, (d) PIL-TFSI-10, (e) PIL-TFSI-20, (f) PIL-TFSI-30, respectively.

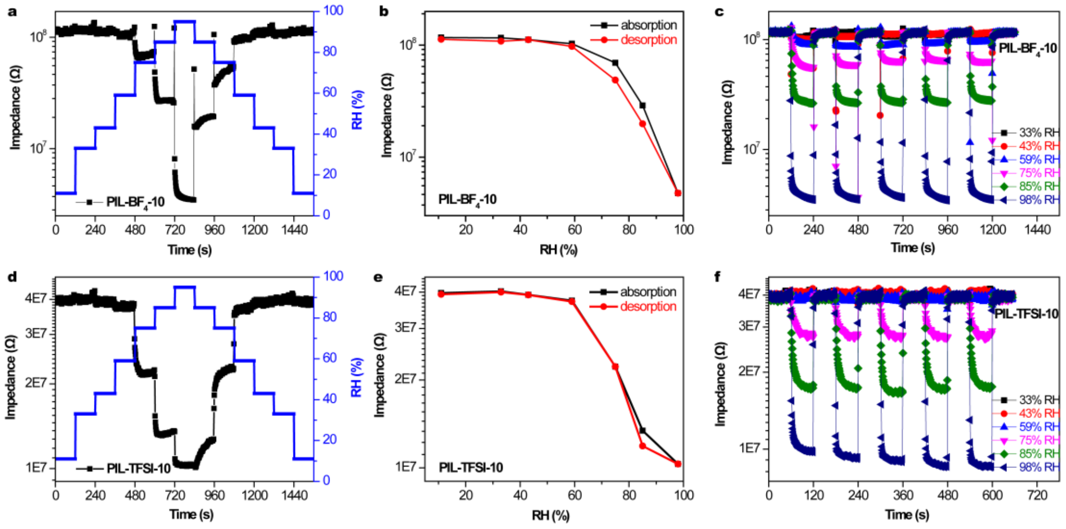

Figure S4. Thickness-dependent performances of PIL humidity sensors: the dynamic response of PIL sensors as the RH increased from 11% to 98% RH and returned to the initial state: (a) PIL-BF_4-10 and (d) PIL-TFSI-10, respectively; the humidity hysteresis of (b) PIL-BF_4-10 and (e) PIL-TFSI-10, respectively; The repeatability characteristic of PIL sensors from 11% to 98% RH: (c) PIL-BF_4-10 and (f) PIL-TFSI-10, respectively.

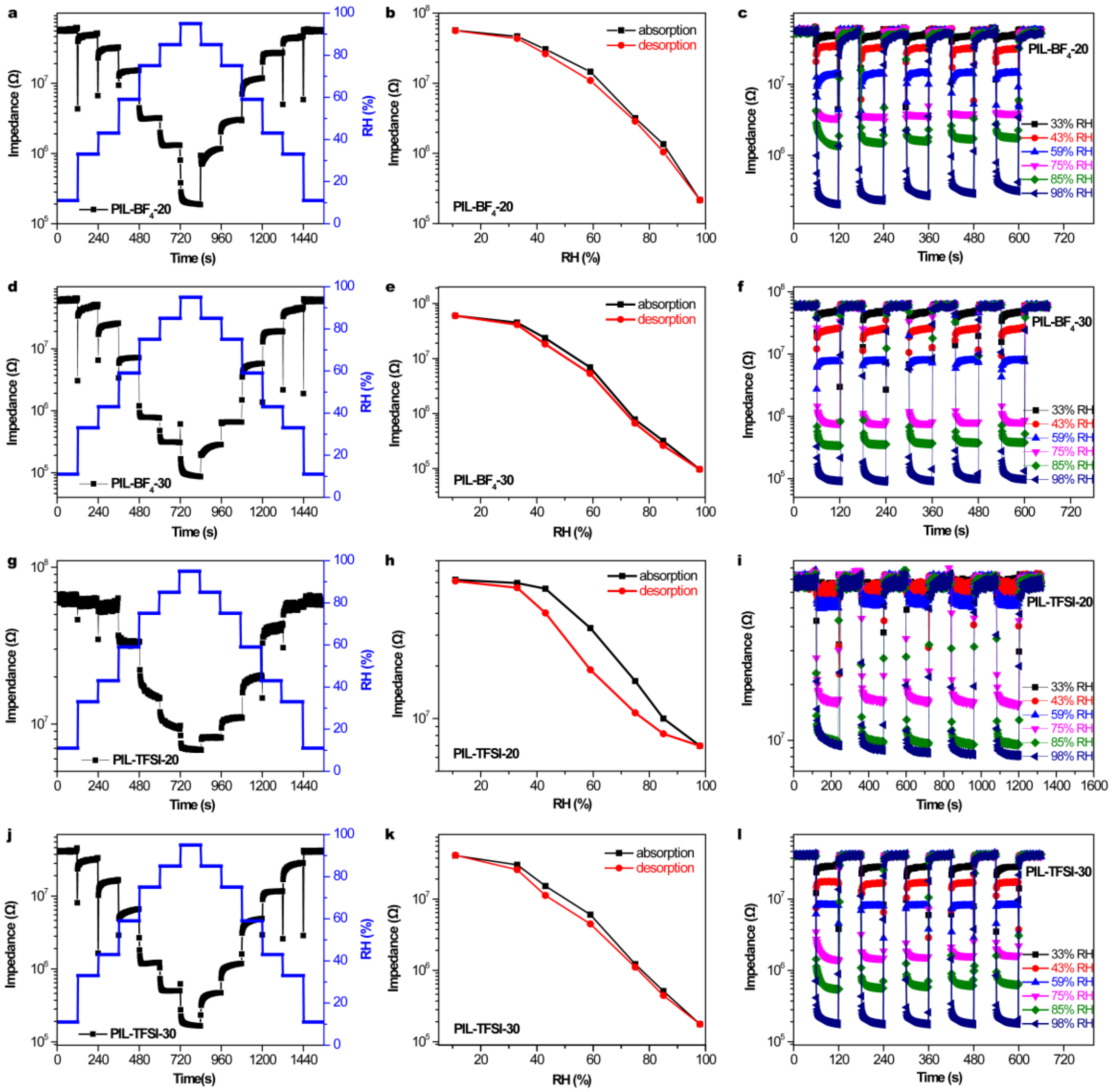

Figure S5. Thickness-dependent performances of PIL humidity sensors: the dynamic response of PIL sensors as the RH increased from 11% to 98% RH and returned to the initial state: (a) PIL-BF_4-20, (d) PIL-BF_4-30, (g) PIL-TFSI-20 and (j) PIL-TFSI-30, respectively; the humidity hysteresis of (b) PIL-BF_4-20, (e) PIL-BF_4-30, (h) PIL-TFSI-20 and (k) PIL-TFSI-30, respectively; The repeatability characteristic of PIL sensors from 11% to 98% RH: (c) PIL-BF_4-20, (f) PIL-BF_4-30, (i) PIL-TFSI-20 and (l) PIL-TFSI-30, respectively.

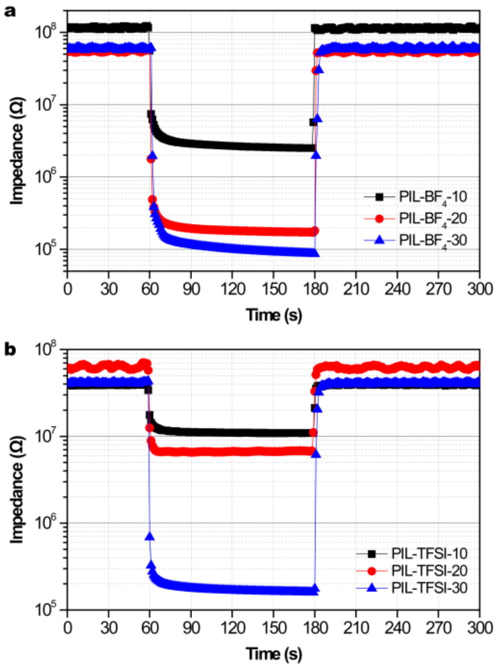

Figure S6. Response and recovery properties of (a) PIL-BF_4 and (b) PIL-TFSI humidity sensors with different thickness.

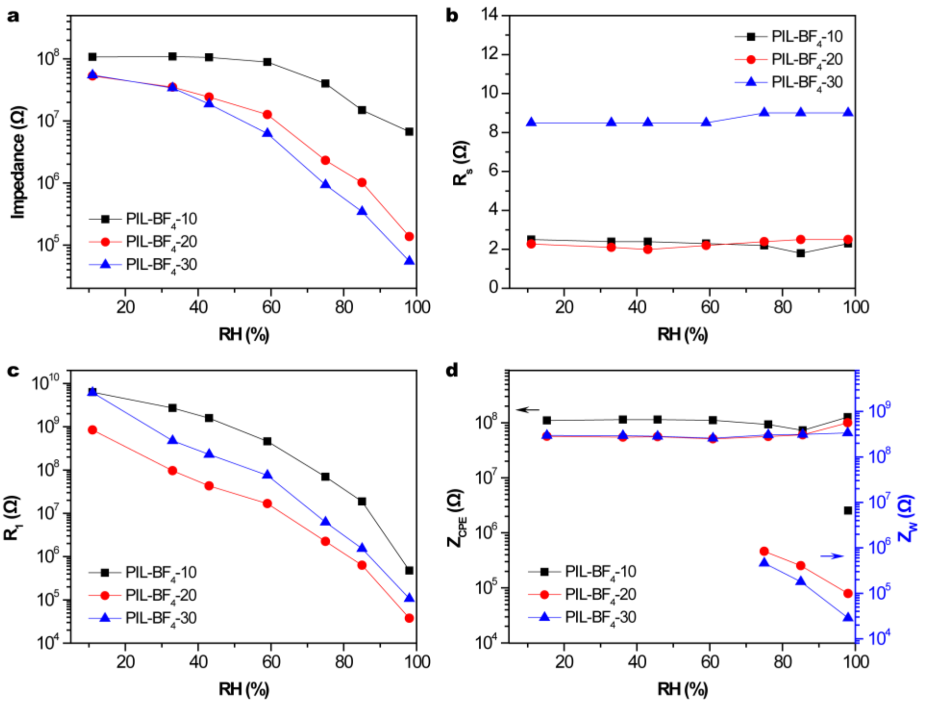

Figure S7. Dependence of the values of (a) total, (b) R_S, (c) R_1 and (d) Z_{CPE} and Z_W on different RH levels for PIL-BF_4 sensors with different thickness.

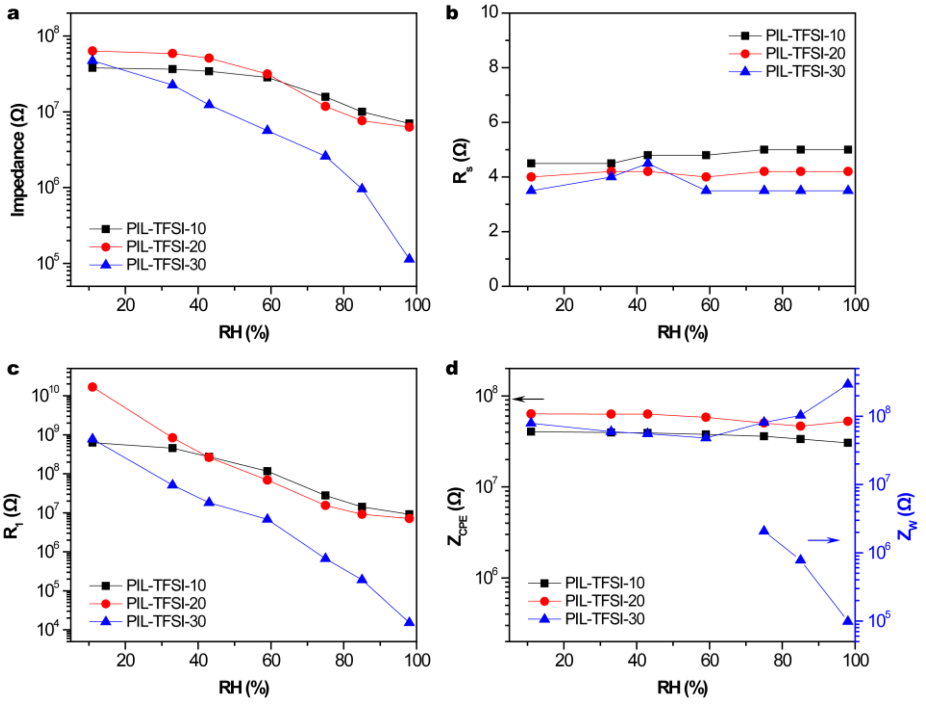

Figure S8. Dependence of the values of (a) total, (b) R_S, (c) R_1 and (d) Z_{CPE} and Z_W on different RH levels for PIL-TFSI sensors with different thickness.

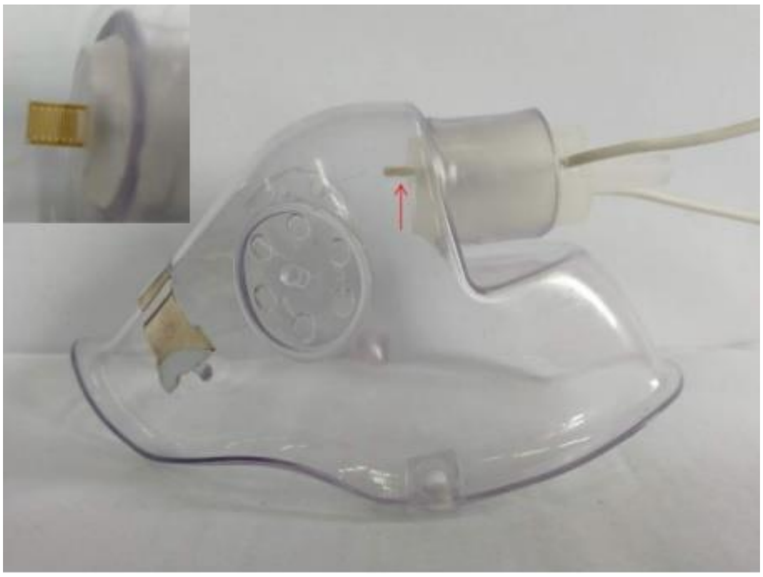

Figure S9. The photo of the breath mask

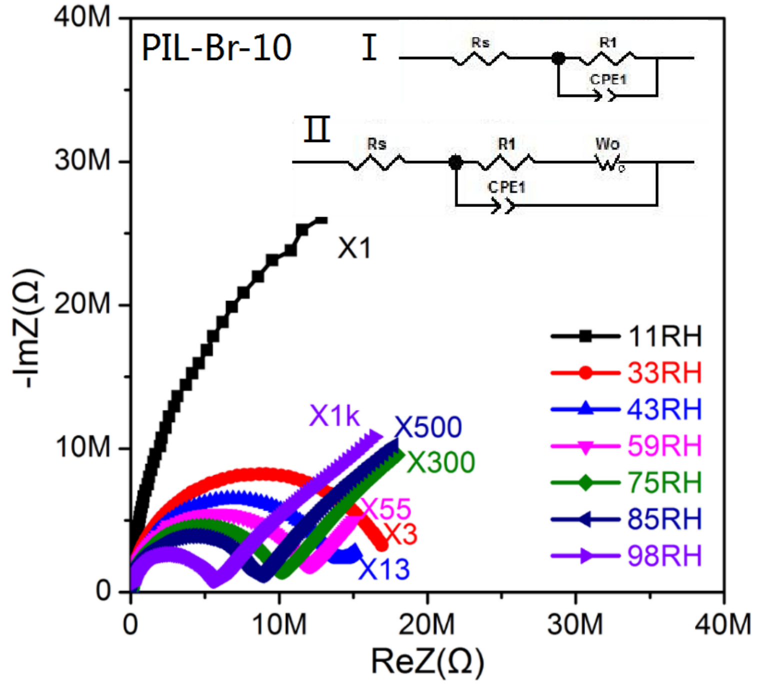

Figure S10. The complex impedance property of the humidity sensor based on PIL-Br-10 in various RH.

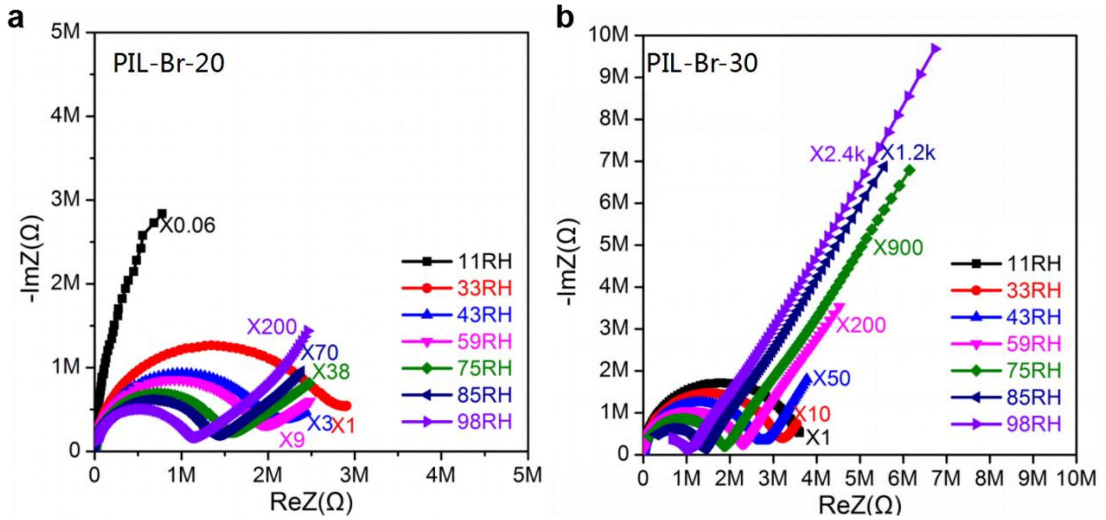

Figure S11. The complex impedance property of humidity sensor based on (a)PIL-Br-20 and (b)PIL-Br-30 in various RH.
